# The Role of the Oral Microbiome and Dental Caries in Respiratory Health: A Systematic Review

**DOI:** 10.3390/jcm14217670

**Published:** 2025-10-29

**Authors:** Łukasz Zygmunt, Sylwia Kiryk, Kamil Wesołek, Jan Kiryk, Izabela Nawrot-Hadzik, Zbigniew Rybak, Klaudia Sztyler, Agata Małyszek, Jacek Matys, Maciej Dobrzyński

**Affiliations:** 1 Faculty of Medicine, Wroclaw University of Science and Technology, building C-7, pl. Grunwaldzki 11, 50-377 Wroclaw, Poland; 2 Department of Pediatric Dentistry and Preclinical Dentistry, Wroclaw Medical University, Krakowska 26, 50-425 Wroclaw, Poland; s.roguzinska@gmail.com (S.K.); klaudia.sztyler@umw.edu.pl (K.S.); maciej.dobrzynski@umw.edu.pl (M.D.); 3 Józef Struś Multi-Specialty Municipal Hospital with a Care and Treatment Facility, Independent Public Health Care Facility, Szwajcarska 3, 61-285 Poznan, Poland; wesolekwesolek@o2.pl; 4 Dental Surgery Department, Wroclaw Medical University, Krakowska 26, 50-425 Wroclaw, Poland; jan.kiryk@umw.edu.pl; 5 Department of Pharmaceutical Biology and Biotechnology, Faculty of Pharmacy, Wroclaw Medical University, 50-556 Wroclaw, Poland; izabela.nawrot-hadzik@umw.edu.pl; 6 Pre-Clinical Research Centre, Wroclaw Medical University, Bujwida 44, 50-345 Wroclaw, Poland; zbigniew.rybak@umw.edu.pl; 7 Department of Biostructure and Animal Physiology, Wrocław University of Environmental and Life Sciences, Kożuchowska 1, 51-631 Wrocław, Poland; agata.malyszek@upwr.edu.pl

**Keywords:** oral microbiome, dental caries, respiratory infection, pneumonia, ventilator-associated pneumonia, oral hygiene

## Abstract

**Objectives:** This systematic review aimed to evaluate the association between oral health—particularly dental caries and dysbiosis of the oral microbiome—and respiratory diseases across different age groups and clinical settings, with emphasis on microbial overlap, clinical outcomes, and preventive strategies. **Methods:** A systematic search was conducted in PubMed, Scopus, Embase, Web of Science, and the Cochrane Library up to June 2025. Eligible studies included randomized controlled trials, cohort, case–control, and cross-sectional investigations examining the relationship between oral diseases or microbiome alterations and respiratory outcomes. Data on study design, population, oral health parameters, microbial taxa, and respiratory endpoints were extracted. Study quality was assessed using the Mixed Methods Appraisal Tool (MMAT, 2018). **Results:** Twenty studies met the inclusion criteria, encompassing pediatric, adult, and elderly populations. Poor oral health, reflected by higher caries indices and periodontal inflammation, was consistently associated with increased risk of lower respiratory tract infections (LRTI), aspiration events, ventilator-associated pneumonia (VAP), and impaired pulmonary function. Oral microbiome analyses revealed enrichment of Veillonella, Prevotella, Klebsiella, and Pseudomonas species in both oral and airway samples, supporting the oral cavity as a reservoir for respiratory pathogens. Interventional evidence from intensive care and nursing home settings demonstrated that structured oral care—particularly daily toothbrushing and chlorhexidine-based plaque control—significantly reduced pneumonia incidence. **Conclusions:** This review confirms a clinically relevant and biologically plausible link between oral dysbiosis, dental caries, and respiratory disease. Oral biofilms contribute to infection risk through microaspiration and microbial seeding of the lower airways. Integrating oral screening, hygiene maintenance, and treatment of active oral disease into respiratory care pathways may reduce respiratory morbidity and mortality, particularly among high-risk populations such as ICU patients, older adults, and individuals with chronic lung disease.

## 1. Introduction

**Figure 1:**
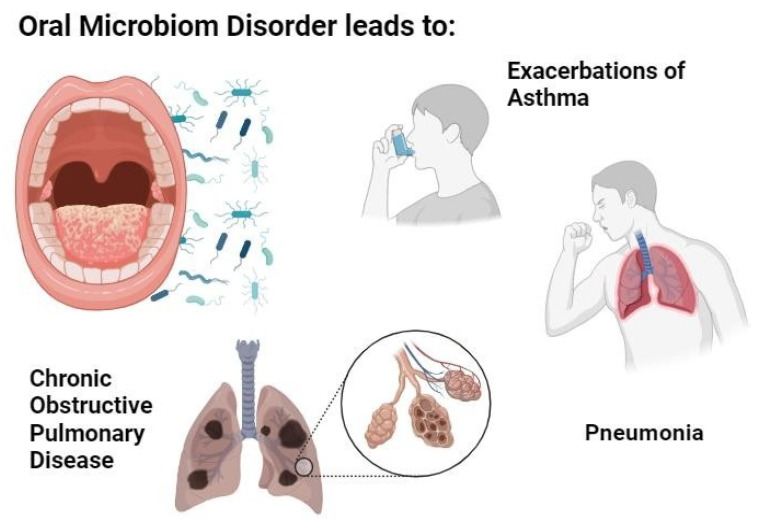
Potential consequences of oral microbiome dysbiosis for the respiratory system.

The oral cavity harbors one of the most complex and diverse microbial ecosystems in the human body, comprising bacteria, fungi, viruses, and archaea that exist in a dynamic state of symbiosis with the host [1,2]. This community performs essential physiological functions, including nutrient metabolism, maintenance of oral pH, modulation of mucosal immunity, biofilm formation, and protection against opportunistic pathogens [3,4]. A disruption of this microbial balance—known as dysbiosis—can promote the proliferation of acidogenic and pathogenic species, leading to oral diseases such as dental caries and periodontitis [5,6,7,8,9,10]. Emerging evidence indicates that dysbiotic oral environments may also act as reservoirs for respiratory pathogens and inflammatory mediators, thereby predisposing individuals to lower respiratory tract infections, including pneumonia, chronic obstructive pulmonary disease (COPD), and asthma exacerbations [11,12,13,14,15,16]. Considering the global burden of both oral and respiratory diseases (according to WHO 1.5 B caries cases and 2.6 M pneumonia deaths annually [17]), understanding how oral microbiome alterations and caries development influence respiratory health has become a critical medical and public health priority [14,18] (Figure 1).

Advances in microbiome analysis techniques have substantially deepened our understanding of the oral–respiratory health axis [19]. Traditional culture-based methods, while valuable for identifying well-known pathogens such as Streptococcus mutans, S. pneumoniae, and Haemophilus influenzae, are limited by low sensitivity and their inability to detect uncultivable microorganisms [2,20,21,22,23]. The introduction of high-throughput molecular approaches—including 16S rRNA gene sequencing, shotgun metagenomics, metatranscriptomics, and metabolomic profiling—has enabled comprehensive, species-level characterization of the oral microbiota and its functional activity [18,19,20]. These advanced methods have revealed distinct dysbiotic patterns associated with dental caries development and with microbial colonization of the lower airways, including increased abundance of Streptococcus, Prevotella, and Veillonella species implicated in respiratory inflammation [5,6,7,15]. Nevertheless, the translation of microbiome profiling into clinical practice remains challenging due to considerable inter-individual variability, temporal dynamics of the microbiome, and confounding environmental or behavioral factors [1,6,24].

An important consideration in this context is the role of dental caries and other oral diseases as potentially modifiable contributors to respiratory disease risk [11]. Carious lesions, periodontal pockets, and oral biofilms can serve as reservoirs of pathogenic microorganisms and host-derived inflammatory mediators that may reach the respiratory tract via aspiration or hematogenous dissemination [12,25,26]. Numerous epidemiological studies have reported associations between poor oral hygiene or untreated dental caries and an increased incidence of respiratory infections—particularly among elderly, immunocompromised, and institutionalized populations [3,21,27,28]. Interventional trials improving oral hygiene in nursing home residents have shown significant reductions in aspiration pneumonia incidence [15,20]. However, the heterogeneity of study designs, outcome measures, and intervention protocols limits generalizability, highlighting the need for a comprehensive and systematic synthesis of available evidence [27,29].

Preventive and therapeutic strategies targeting the oral microbiome to mitigate both dental and respiratory risks are increasingly recognized [7]. Core preventive measures—such as mechanical plaque control, topical fluoride application, and regular professional prophylaxis—remain fundamental for maintaining oral health [6]. Adjunctive microbiome-modulating interventions, including probiotics, prebiotics, synbiotics, and antimicrobial mouthrinses (e.g., chlorhexidine), have demonstrated potential in restoring microbial homeostasis and reducing pathogenic colonization [6]. Nonetheless, uncertainties persist regarding their long-term efficacy, patient compliance, adverse effects, and the risk of microbial resistance. Emerging approaches based on precision microbiomics and personalized oral care offer promising prospects, yet they require further validation in well-designed clinical studies.

Building upon the growing body of evidence, this systematic review aims to comprehensively assess the role of the oral microbiome and dental caries in respiratory health. Specifically, it seeks to answer the research question: How do alterations in the oral microbiome and the presence of dental caries influence the risk and progression of respiratory diseases? To address this, we integrate findings from observational and interventional studies to identify shared microbiological and clinical mechanisms linking oral and respiratory conditions. To the best of the authors’ knowledge, no previous systematic review has comprehensively synthesized evidence on this topic, unlike the 2020 periodontitis-focused review [30], underscoring the novelty and relevance of the present work in guiding interdisciplinary strategies for integrating oral and respiratory health.

## 2. Materials and Methods

### 2.1. Focused Question

The systematic review followed the PICO framework as follows [31].

PICO question: In patients with respiratory diseases (Population), does the presence of oral diseases—particularly dental caries and oral microbiome dysbiosis—(Exposure) affect the incidence, severity, or clinical course of respiratory conditions (Outcome) compared with individuals presenting healthy oral status (Comparison)?

### 2.2. Protocol

**Figure 2:**
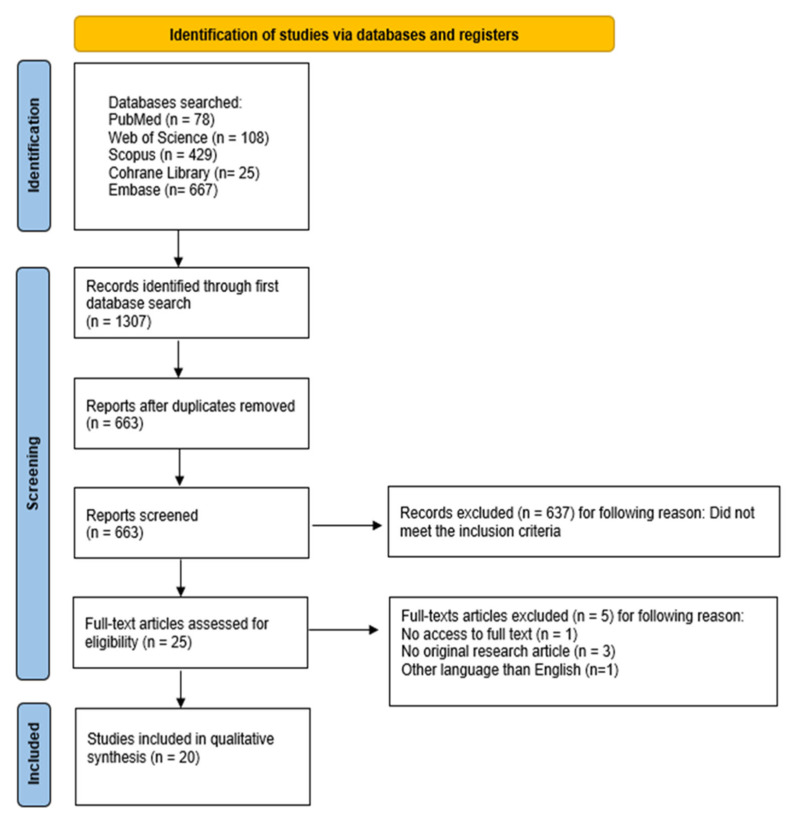
The PRISMA 2020 flow diagram [34].

The article selection process for this systematic review was conducted in accordance with the PRISMA 2020 guidelines and is presented in the corresponding flow diagram (Figure 2) [32,33,34,35,36,37,38,39,40]. The review protocol was prospectively registered on the Open Science Framework (OSF) under the following link: https://osf.io/4gh2v/ (accessed on 7 October 2025).

### 2.3. Eligibility Criteria

Studies were deemed eligible for inclusion if they fulfilled the following conditions:

- Investigated associations between oral and respiratory diseases;
- Were published in English;
- Represented prospective case series, non-randomized controlled studies (NRS), or randomized controlled trials (RCTs).

The exclusion criteria were defined as follows:

- Lack of a clear association between oral and respiratory conditions;
- Non-English language publications;
- Case or clinical reports;
- Opinion papers, editorials, or narrative reviews;
- Absence of full-text availability;
- Duplicate publications.

No publication year restrictions were applied.

### 2.4. Information Sources, Search Strategy, and Study Selection

A comprehensive literature search was performed in June 2025 across five electronic databases: PubMed, Scopus, Cochrane Library, Embase, and Web of Science (WoS). The search aimed to identify studies that fulfilled the predefined inclusion criteria. To specifically capture research addressing the relationship between oral and respiratory diseases, searches were limited to titles, abstracts, and keywords. The following combination of search terms and Boolean operators was applied: (‘caries’ OR ‘dental caries’ OR ‘tooth decay’) AND (‘pulmonary disease’ OR ‘COPD’ OR ‘asthma’ OR ‘pneumonia’ OR ‘obstructive apnea’ OR ‘lung diseases’ OR ‘oral-lung axis’ OR ‘respiratory tract’) AND (‘microbiome’ OR ‘oral microbiome’ OR ‘microbiota’ OR ‘bacteria’ OR ‘virus’ OR ‘fungi’).

Only studies with available full-texts and published in English were considered eligible. All searches adhered to the previously defined eligibility criteria. The exact database-specific search strategies were as follows:

PubMed: (“caries” OR “dental caries” OR “tooth decay”) AND (“pulmonary disease” OR “COPD” OR “asthma” OR “pneumonia” OR “obstructive apnea” OR “lung diseases” OR “oral-lung axis” OR “respiratory tract”) AND (“microbiome” OR “oral microbiome” OR “microbiota” OR “bacteria” OR “virus” OR “fungi”)

Scopus: TITLE-ABS-KEY ((“caries” OR “dental caries” OR “tooth decay”) AND (“pulmonary disease” OR “COPD” OR “asthma” OR “pneumonia” OR “obstructive apnea” OR “lung diseases” OR “oral-lung axis” OR “respiratory tract”) AND (“microbiome” OR “oral microbiome” OR “microbiota” OR “bacteria” OR “virus” OR “fungi”))

Web of Science (WoS): TS = ((“caries” OR “dental caries” OR “tooth decay”) AND (“pulmonary disease” OR “COPD” OR “asthma” OR “pneumonia” OR “obstructive apnea” OR “lung diseases” OR “oral-lung axis” OR “respiratory tract”) AND (“microbiome” OR “oral microbiome” OR “microbiota” OR “bacteria” OR “virus” OR “fungi”))

Embase: (‘caries’ OR ‘dental caries’ OR ‘tooth decay’) AND (‘pulmonary disease’ OR ‘COPD’ OR ‘asthma’ OR ‘pneumonia’ OR ‘obstructive apnea’ OR ‘lung diseases’ OR ‘oral-lung axis’ OR ‘respiratory tract’) AND (‘microbiome’ OR ‘oral microbiome’ OR ‘microbiota’ OR ‘bacteria’ OR ‘virus’ OR ‘fungi’)

Cochrane Library: (“caries” OR “dental caries” OR “tooth decay”) AND (“pulmonary disease” OR “COPD” OR “asthma” OR “pneumonia” OR “obstructive apnea” OR “lung diseases” OR “oral-lung axis” OR “respiratory tract”) AND (“microbiome” OR “oral microbiome” OR “microbiota” OR “bacteria” OR “virus” OR “fungi”)

### 2.5. Data Collection Process and, Data Items

The studies that met the inclusion criteria were independently reviewed and extracted by four authors (J.K., S.K., Ł.Z. and K.W.). The extracted information included the first author’s name, year of publication, study design, article title, details of study group, and microbiological outcome. All relevant data were systematically recorded in a standardized Excel spreadsheet.

### 2.6. Risk of Bias Assessment in Individual Studies

During the initial phase of study selection, each reviewer independently screened the titles and abstracts to minimize potential selection bias. The Cohen’s kappa coefficient was applied to evaluate the level of inter-reviewer agreement. Any disagreements regarding study inclusion or exclusion were resolved through discussion and consensus among the authors [41].

### 2.7. Quality Assessment

Two independent reviewers (J.M. and M.D.), blinded to each other’s evaluations, assessed the methodological quality of all included studies using the Mixed Methods Appraisal Tool (MMAT), version 2018. This validated instrument enables a consistent evaluation of diverse study designs, including randomized, non-randomized, and descriptive quantitative studies, as well as mixed-methods research. The MMAT comprises five core criteria designed to appraise methodological coherence, integration of study components, and overall validity.

The following guiding questions were used in the appraisal process:

1 Is there an adequate rationale for using a mixed-methods design to address the research question?
2 Are the different components of the study effectively integrated to answer the research question?
3 Are the outputs of the integration of qualitative and quantitative components adequately interpreted?
4 Are divergences and inconsistencies between quantitative and qualitative results adequately addressed?
5 Do the different components of the study adhere to the quality criteria of each methodological tradition involved?

Each criterion was rated as “Yes,” “No,” or “Can’t tell.” Discrepancies between reviewers were resolved through discussion until consensus was achieved. Inter-rater reliability was calculated using Cohen’s kappa coefficient with MedCalc software (version 23.1.7; MedCalc Software Ltd., Brussels, Belgium). The resulting κ value of 0.81 (*p* < 0.001) indicated a high level of agreement, reflecting near-perfect consistency between the reviewers.

## 3. Results

### 3.1. Study Selection

The systematic search conducted across five electronic databases—PubMed, Scopus, Cochrane Library, Embase, and Web of Science—identified a total of 1307 records. After removal of duplicates and initial screening of titles and abstracts, 663 unique articles were retained for detailed review. Only original research studies comparing at least two groups were considered eligible, whereas case reports, editorials, letters, book chapters, and non-English publications were excluded. Following a three-stage screening process, 25 studies initially fulfilled the inclusion criteria. Subsequently, five articles were excluded due to lack of full-text access, non-English language, or case report design, resulting in 20 studies being included in the final synthesis. The study selection process is summarized in the PRISMA flow diagram (Figure 2). The characteristics of the included studies are presented in Table 1, while Supplementary Table S1 provides an overview of the main findings and associations between oral health parameters and respiratory outcomes.

### 3.2. Characteristics of the Included Studies

A total of 20 studies were included in the final analysis, encompassing a wide range of research designs—randomized controlled trials (RCTs, *n* = 2 [49,56]), prospective or retrospective cohort studies (*n* = 5 [18,42,43,47,50]), case–control and cross-sectional investigations (*n* = 10 [14,44,45,52,53,54,55,59,60,61]), and laboratory or case-series analyses (*n* = 3 [48,51]). Collectively, these studies explored the relationship between oral health status, microbial dysbiosis, and respiratory outcomes across pediatric, adult, and elderly populations in various settings, including community samples, hospitals, and intensive care units.

Several studies focused on pediatric and adolescent populations, showing that poorer oral hygiene, higher caries experience, or distinct oral microbiome profiles were associated with an increased risk or altered course of respiratory conditions such as asthma, allergies, and recurrent respiratory tract infections [12,18,44,55,59]. In adults and older patients, cohort and cross-sectional studies reported significant associations between periodontitis, dental caries, or oral microbiome imbalance and adverse respiratory outcomes, including chronic obstructive pulmonary disease (COPD), postoperative pneumonia, aspiration pneumonia, and nosocomial infections [14,42,43,45,47,52,53,54,58,60].

Two randomized clinical trials [56,60] provided interventional evidence that structured dental care and chlorhexidine-based plaque control protocols in intensive-care settings significantly reduced the incidence of lower respiratory tract infections, particularly ventilator-associated pneumonia (VAP). Microbiological and laboratory studies [48,51] further demonstrated the presence of pathogenic and antibiotic-resistant microorganisms—such as *Klebsiella pneumoniae*, *Staphylococcus aureus*, and *Candida albicans*—in oral lesions or odontogenic sinus infections, underscoring the role of the oral cavity as a potential reservoir for respiratory pathogens [62].

Most studies (approximately three-quarters [14,18,42,43,44,45,47,50,51,52,53,54,55,56,58,59,60]) reported positive associations between impaired oral health or dysbiosis of the oral microbiome and increased respiratory morbidity. A minority of studies found null or inverse relationships, such as a lower incidence of upper respiratory tract infections among children with higher caries indices [18]. The detailed characteristics of each study, including design, population, and principal findings, are presented in Table 1.

### 3.3. Main Findings

Across the included studies, several consistent associations emerged between oral pathology, microbial dysbiosis, and respiratory outcomes (see Supplementary Table S1). The principal findings are summarized as follows:

#### 3.3.1. Poor Oral Health and Increased Risk of Respiratory Infections

Several studies consistently demonstrated that active oral disease, including caries and periodontitis, was associated with higher respiratory morbidity. Active dental caries emerged as an independent risk factor for postoperative pneumonia in thoracic surgery patients [43]. The severity of periodontitis was significantly correlated with reduced pulmonary function (lower FEV_1_) [53]. In children, early-life lower respiratory tract infections (LRTI) predicted greater caries experience in permanent teeth during adulthood, suggesting a bidirectional association between oral and respiratory health [50]. Pulmonary patients also exhibited worse periodontal status, halitosis, and xerostomia than controls [45]. Poor oral health may exacerbate respiratory outcomes and contribute to chronic, reciprocal interactions between dental and pulmonary diseases.

#### 3.3.2. Oral Microbiome Dysbiosis as a Reservoir for Respiratory Pathogens

Multiple studies revealed substantial alterations in the oral microbiota among patients at increased respiratory risk. In asthmatic and allergic children, dental biofilm showed enrichment of *Capnocytophaga*, *Fusobacterium*, and *Prevotella_6* [55], while caries-affected asthmatic children exhibited increased *Veillonella* and *Prevotella* species linked to respiratory pathogens [59]. In long-term care patients fed via nasogastric tubes, the oral microbiome was enriched with *Pseudomonas*, *Corynebacterium*, and *Fusobacterium*, whereas beneficial genera such as *Streptococcus* and *Veillonella* were depleted—changes correlated with a higher prevalence of pneumonia [49,60]. Similarly, cystic fibrosis patients presented lower oral microbial diversity with increased *Candida* and respiratory-associated taxa [42]. Oral dysbiosis may facilitate colonization by opportunistic pathogens relevant to respiratory infections, supporting the concept of the oral cavity as a microbial reservoir.

#### 3.3.3. Periodontal Disease and Lung Disease: Microbiological and Clinical Link

Men with severe periodontitis showed increased abundance of *Porphyromonas* species and reduced microbial diversity, correlating with impaired lung function [53]. Thoracic surgery patients with untreated caries and periodontitis were more likely to develop postoperative pneumonia [43], while individuals with chronic pulmonary diseases exhibited more advanced periodontal destruction than healthy controls [45]. Periodontal inflammation and dysbiosis appear to be both clinical and microbiological risk factors for declining lung function and recurrent respiratory infections.

#### 3.3.4. Pharmacotherapy for Respiratory Diseases and Its Impact on the Oral Microbiome and Caries Development

Asthma and COPD patients using inhaled corticosteroids and bronchodilators showed higher caries indices (DMFT/DMFS), greater loads of cariogenic and opportunistic organisms (*Streptococcus mutans*, *Lactobacillus casei*, *Candida albicans*), and reduced salivary flow [14]. Similar findings were observed among adolescents with asthma undergoing long-term inhaled therapy, who demonstrated greater caries prevalence and microbial imbalance [44]. Long-term inhaled medications can alter salivary physiology and microbiome composition, promoting caries development and reinforcing the oral–lung connection.

#### 3.3.5. Shared Colonization Between the Oral Cavity and Respiratory Tract

Direct evidence of pathogen overlap between the oral cavity and lower airways was reported in several studies. In intensive care patients, identical bacterial strains were identified in dental plaque and tracheal aspirates [60]. Thoracic surgery patients developed pneumonia caused by organisms previously isolated from oral samples [43], while elderly individuals with dysphagia exhibited high oral and nasopharyngeal colonization with *Klebsiella* and *Pseudomonas*, frequently leading to aspiration [54]. These findings confirm cross-colonization, whereby oral pathogens can directly seed the respiratory tract, contributing to infection risk.

#### 3.3.6. Oral Hygiene Interventions Reduce Respiratory Infection Risk

Interventional and preventive studies consistently showed that improved oral hygiene decreases the incidence of lower respiratory infections. In ICU settings, professional dental care significantly reduced LRTI and VAP incidence (relative risk ≈ 0.44) [56], while 0.2% chlorhexidine gel application reduced plaque colonization by hospital pathogens [49]. Additionally, regular oral decontamination was associated with fewer shared strains between dental plaque and tracheal samples [60]. Systematic oral care, including mechanical hygiene and antiseptic regimens, effectively reduces nosocomial pneumonia and VAP, emphasizing its role in respiratory infection prevention.

#### 3.3.7. Hospital and ICU Outcomes

Two randomized trials [49,56] confirmed that structured dental care and chlorhexidine plaque control significantly reduced VAP and LRTI incidence in ICU patients. Observational data further validated dental plaque as a microbial reservoir [60] and identified active caries as a predictor of postoperative pneumonia [43]. These findings highlight that integrating oral hygiene protocols into hospital and ICU care pathways yields measurable reductions in respiratory complications.

### 3.4. Quality Assessment

**Figure 3:**
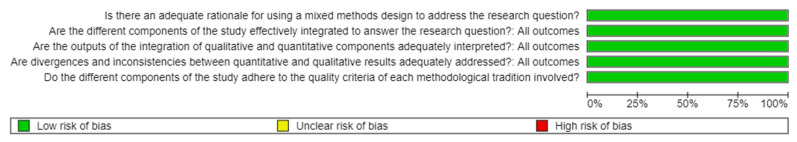
Quality assessment.

All qualified publications received the maximum number of points and were assessed as high quality (see Figure 3 and Supplementary Table S2).

## 4. Discussion

Our systematic review aimed to investigate the association between oral diseases—particularly dental caries and dysbiosis of the oral microbiome—and adverse respiratory outcomes across diverse clinical settings. By synthesizing findings from 20 primary studies, we observed that poorer oral health, reflected by higher DMFT/dmft indices, greater plaque accumulation, and periodontal inflammation, was consistently associated with lower respiratory tract infections (LRTI), aspiration events, ventilator-associated pneumonia (VAP), and impaired pulmonary function [14,18,42,43,44,45,46,47,48,49,50,51,52,53,54,55,56,58,59,60]. These results support the growing body of evidence suggesting that the lower airways are continuously exposed to supraglottic microbiota through microaspiration, with oral biofilms serving as persistent reservoirs that modulate both pulmonary immunity and infection susceptibility. Notably, one pediatric cohort [18] reported an inverse association between upper respiratory tract infections (URTI) and caries experience, highlighting potential age-related, immunological, or environmental modifiers that warrant further investigation. By emphasizing caries-associated taxa and clinical phenotypes—including ICU patients, the elderly, individuals with asthma or COPD, and those undergoing perioperative care—our review reinforces the concept of dynamic microbial exchange between the oral cavity and the lower respiratory tract. These findings expand upon prior work demonstrating the oropharyngeal origin of the lung microbiome and its relevance in respiratory pathophysiology [63,64,65,66,67,68,69].

When compared with previous research, our synthesis indicates a strong and consistent association between dental caries and tooth loss and an increased risk of pneumonia, hospitalization, and respiratory-related mortality [14,43,45,49,50,53,54,57,58,60,70,71,72]. This relationship was observed across different age groups and clinical settings, although its strength varied depending on population characteristics and data collection methods. Population-based studies have demonstrated that greater caries experience and tooth loss are linked to a higher incidence of pneumonia, whereas regular toothbrushing and professional dental cleaning exert a protective effect. These findings align with our results showing that active caries and poor oral hygiene were significant predictors of postoperative pneumonia and lower respiratory tract infections (LRTI) [43,58,60,72]. Evidence from pediatric populations also suggests that early childhood caries may precede recurrent upper respiratory tract infections (URTI) or prolonged cough, consistent with our observation that early-life respiratory infections predict future caries burden [11,13,50,70,71]. Nonetheless, one cohort study [18] reported an inverse association between caries and URTI frequency, likely influenced by confounding factors such as healthcare-seeking behavior, antibiotic exposure, or socioeconomic differences. Taken together, the evidence supports a clinically relevant, bidirectional association between oral and respiratory health—where dental caries and poor oral hygiene increase the risk of respiratory morbidity, and chronic respiratory conditions are often accompanied by worse oral health indicators. These conclusions are in line with large cohort and mortality analyses linking oral disease to pneumonia and respiratory outcomes [70,71,72].

Microbiome-resolved findings from our review reinforce the biological plausibility of the oral–respiratory axis by demonstrating a clear overlap between cariogenic and periodontal microbial communities and lower-airway microbiota profiles. Across both asthma/allergy cohorts and intensive care settings, a recurring enrichment of *Veillonella* and *Prevotella* species was observed in oral biofilms, together with opportunistic pathogens such as *Klebsiella* and *Pseudomonas* in high-risk hosts [42,47,49,52,54,55,58,59,60]. This pattern mirrors evidence from airway microbiome studies showing that the lower respiratory tract frequently harbors supraglottic taxa introduced through microaspiration. Moreover, the predominance of oral-origin taxa has been linked to Th17-skewed inflammation, heightened disease activity, and airway remodeling in asthma and COPD [63,64,65,66,67,68,69,70,73,74,75,76,77,78,79]. Even studies utilizing bronchoalveolar lavage and lung tissue samples—while controlling for upper-airway contamination—have consistently detected oral and nasopharyngeal genera in situ, lending support to the aspiration-seeding model that underpins our clinical synthesis [65,73,80]. Our review also underscores the importance of disease-specific modifiers influencing this relationship. For example, patients with cystic fibrosis exhibit reduced oral bacterial diversity and increased *Candida* colonization [42,81], whereas tube-fed or dysphagic individuals demonstrate oral enrichment with Gram-negative aerobes resembling aspiration pathogens [47,54]. These patterns are consistent with respiratory-focused microbiome research linking oropharyngeal taxa to clinical phenotype, infection susceptibility, and disease outcomes [74,82,83,84].

From a clinical perspective, our findings highlight the importance of implementing practical and cost-effective oral health interventions across high-risk patient populations. In intensive care unit (ICU) settings, structured oral hygiene protocols and dental involvement can substantially reduce lower respiratory tract infections (LRTI) and ventilator-associated pneumonia (VAP) by 34% (RR 0.66; 95% CI 0.56–0.77; *n* = 12,500; GRADE high certainty) without evidence of harm [49,56,60,85,86]. External data further corroborate these findings, showing that routine toothbrushing—independent of chlorhexidine use—lowers the incidence of hospital-acquired pneumonia and ICU mortality, with additional benefits for the duration of mechanical ventilation and length of ICU stay [70,76,77,78,79]. In long-term care and nursing home environments, comprehensive mouth-care programs have been shown to decrease pneumonia rates in the short term, although sustained adherence is necessary to preserve their effectiveness. This observation aligns with our synthesis of elderly and dysphagic cohorts, where poor oral hygiene and pathogen-rich biofilms were linked to aspiration and pneumonia risk [54,75]. Similarly, among perioperative patients, the treatment of active caries and optimization of oral hygiene may mitigate postoperative pulmonary complications, as observed in our included thoracic surgery cohort [43]. Emerging evidence also suggests that periodontal therapy can improve small-airway function and reduce airway inflammation, supporting the integration of oral health assessment and management into respiratory care bundles and prehabilitation pathways [52,87,88,89,90]. These findings are particularly relevant to antimicrobial stewardship, as oral reservoirs of resistant organisms—including methicillin-resistant *Staphylococcus aureus* (MRSA) and third-generation cephalosporin-resistant *Enterobacterales*—are frequently identified in tube-fed or prosthesis-wearing elderly patients, precisely the populations highlighted in our review [52,54,91].

Several limitations should be acknowledged when interpreting our findings. First, substantial heterogeneity in study designs, diagnostic definitions (both dental and respiratory), and timing of exposure–outcome assessment limits the feasibility of meta-analytic synthesis and increases the risk of residual confounding. Future research should adopt standardized indices for caries and plaque assessment, alongside adjudicated and clearly defined pneumonia outcomes. Second, many microbiome studies remain cross-sectional, underpowered, or vulnerable to upper-airway contamination. Rigorous sampling approaches, including replicate bronchoalveolar lavage (BAL) procedures and source-tracking analyses, are essential to accurately determine the origin, viability, and function of microbial taxa within the lung environment [17,65,66,73]. Third, relatively few interventional trials isolate the specific effects of mechanical plaque control from those of antiseptic agents or bundled preventive measures. Adequately powered, multi-arm randomized controlled trials (RCTs) across intensive care and long-term care settings are therefore needed to clarify causal mechanisms and optimize preventive strategies [30,70,76,77,79]. Fourth, important mechanistic gaps persist. Establishing causal links between discrete oral taxa, microbial metabolites, and airway immune pathways (e.g., γδ-T cell activation, Th17 polarization) will require longitudinal, multi-omics studies that integrate oral–lung profiling with measured aspiration events and medication exposures [74,86]. Our review contributes to this evolving field by identifying a consistent clinical–microbial signature across diverse populations, centered on caries and oral dysbiosis as modifiable co-determinants of respiratory risk. Moving forward, we propose a translational agenda that integrates routine oral screening and daily toothbrushing into respiratory care pathways, evaluates targeted periodontal and caries therapy as part of prehabilitation, and embeds robust microbiome endpoints into future clinical trials. Qianqian He et. al. drew similar conclusions from their research [92]. The unanimity of the researchers supports the introduction of the proposed solution and provides a favorable prospect for its positive adoption as a standard in medical care.

## 5. Conclusions

This systematic review confirms and strengthens the evidence for a consistent and clinically meaningful association between oral health—particularly dental caries and microbial dysbiosis—and adverse respiratory outcomes across diverse populations and care settings. Oral biofilms function as reservoirs for respiratory pathogens, and their translocation through microaspiration contributes to an increased risk of respiratory infections, impaired lung function, and poorer clinical outcomes. Caries- and periodontitis-associated taxa such as *Veillonella*, *Prevotella*, *Klebsiella*, and *Pseudomonas* were frequently shared between the oral cavity and the lower airways, supporting the biological plausibility of the mouth–lung axis. These findings underscore the importance of integrating oral health maintenance into respiratory disease prevention and management—particularly among high-risk groups such as ICU patients, older adults, and individuals with chronic lung conditions. Simple and low-cost measures, including daily toothbrushing, professional dental care, and the treatment of active oral disease, hold promise for reducing respiratory morbidity and mortality. Embedding structured oral health assessments and hygiene protocols within respiratory care pathways represents a feasible and effective strategy to improve clinical outcomes. Future research should focus on longitudinal and interventional studies that combine standardized clinical endpoints with advanced microbiome profiling to clarify causal mechanisms and optimize intervention strategies. Well-designed randomized controlled trials are needed to disentangle the effects of mechanical plaque control from antiseptic agents and to evaluate targeted caries and periodontal therapies in both hospital and community settings. Strengthening collaboration between dental and respiratory disciplines through shared preventive frameworks may yield substantial clinical and public health benefits.

## Figures and Tables

**Table 1:** General characteristics of studies.

Study	Aim of the Study	Materials and Methods	Results	Conclusions
Zhou 2018 [16]	To investigate the association between oral health and upper respiratory tract infection in children.	In 288 children aged 4 years, the DMFT and plaque index were assessed and a history of upper respiratory tract infections in the last 12 months was collected.	A significantly higher rate of dmft was observed in children who had no upper respiratory tract infections compared to children who had at least 3 infections.	Children with poorer oral health are less likely to experience upper respiratory tract infections.
Winning 2023 [42]	To investigate the relationship between subgingival microbial diversity associated with periodontitis and reduced respiratory function	Subgingival plaque swabs were collected from 507 men and their microbial diversity index (MDI) was assessed, and spirometry was performed.	An increase in MDI by 1 was associated with a 0.71% decrease in FEV1.	The diversity of subgingival microorganisms is associated with reduced respiratory function.
Rantala 2016 [43]	Assessment of the relationship between the occurrence of respiratory infections before the eruption of permanent teeth and the development of dental caries.	Information on the occurrence of respiratory infections was obtained from the medical records of 1623 patients, and then information on the number of dental fillings was obtained from the interview.	The average number of teeth with fillings was 1.4 higher in patients who experienced a respiratory infection requiring hospitalization before the age of 2 years.	The occurrence of respiratory infections in early childhood is associated with poorer condition of the permanent dentition.
Shirazian 2018 [44]	Assessment of oral diseases in patients with lung diseases compared to healthy individuals.	In 42 patients from the respiratory ward and healthy candidates, periodontal status, gingivitis, xerostomia, halitosis, dental caries, tongue coating and existence of oral lesion were assessed.	The incidence of gingivitis, periodontitis, halitosis and tongue coating in patients with lung diseases was significantly higher than in healthy individuals.	More attention needs to be paid to improving the oral health of patients suffering from lung diseases.
Ploenes 2022 [45]	To investigate the relationship between oral health and postoperative pneumonia.	230 patients were assessed for the presence of caries and periodontal disease, number of lost teeth, and regularity of dental visits the day before thoracic surgery.	Patients with a higher frequency of dental caries who do not regularly visit the dentist have a significantly higher incidence of postoperative pneumonia.	Poor oral health is a risk factor for postoperative complications.
Arweiler 2021 [46]	To investigate the difference in salivary biofilm and dental plaque in children with respiratory allergies compared to healthy children.	RNA sequencing studies were performed on material collected from saliva and dental plaque of 46 pediatric patients from the allergy department and healthy candidates.	Bacteria in the saliva of healthy children are more diverse. In the dental plaque of children with allergies, an increase in the number of Fusobacterium nucleatum bacteria was noted compared to healthy children, while the number of Fusobacterium unclassified and Prevotella_6 unclassified decreased.	The study results indicate the possible involvement of dental biofilm in the development of allergies and asthma in children.
Bellissimo-Rodrigues 2014 [41]	To evaluate whether incorporating comprehensive dental care into ICU patients’ oral hygiene regimens can reduce the incidence of nosocomial lower respiratory tract infections (LRTIs), including ventilator-associated pneumonia (VAP).	Design: Observer-blind randomized clinical trial. Setting: General ICU at University Hospital, Ribeirão Preto Medical School, Brazil. Participants: 254 adult patients expected to stay ≥ 48 h in the ICU. Intervention: Experimental group (*n* = 127): Received dental care by a dentist (4–5 times/week), including brushing, tongue scraping, calculus removal, caries treatment, and extractions, plus chlorhexidine. Control group (*n* = 127): Routine oral hygiene by nurses using gauze and chlorhexidine only. Outcomes Measured: Primary: Incidence of LRTI. Secondary: Mortality, antibiotic use, ICU stay duration, adverse events.	LRTI Incidence: Experimental group: 8.7% Control group: 18.1% Adjusted RR: 0.44 (95% CI, 0.20–0.96); *p* = 0.04 VAP Rate: Control: 16.5 per 1000 ventilator-days Experimental: 7.6 per 1000 ventilator-days (*p <* 0.05) Mortality: No significant difference (≈30% in both groups). Adverse Events: Mild (mucosal irritation, minor bleeding); more frequent in the intervention group but not severe.	Comprehensive dental care is safe and effective in preventing LRTIs among ICU patients. The intervention significantly reduces infection rates, particularly VAP. No major complications were associated with dental procedures. Further studies may be needed to assess mortality impact and cost-effectiveness in other settings.
Cherkasov 2019 [47]	To investigate whether differences exist in the dental plaque microbiota of asthmatic children with and without dental caries, using 16S rDNA sequencing.	Design: Observational, cross-sectional study using 16S rDNA sequencing. Participants: 18 asthmatic children (ages 3–6). Group A: 8 children with asthma but no dental caries. Group C: 10 children with asthma and dental caries. Sampling: Dental plaque was collected from the second mandibular left molar (caries-free) or the most affected tooth (caries-active). Analysis: DNA extracted from plaque. Sequencing performed on Illumina MiSeq (targeting V3–V4 region). Bioinformatic analysis included OTU clustering, alpha and beta diversity assessment, and taxonomic classification.	No significant differences in both groups Key microbial findings: Caries-associated (“caries-enriched”) taxa: Veillonella, Prevotella loescheii, Prevotella histicola, Kingella oralis, Haemophilus haemolyticus. Caries-depleted taxon: Neisseria (especially N. oralis). Core microbiota in both groups included Streptococcus, Neisseria, Veillonella, Prevotella, Haemophilus, and Porphyromonas. Several identified microorganisms are potential respiratory pathogens, suggesting overlap between cariogenic and asthma-associated flora.	The overall bacterial diversity and community structure of dental plaques in asthmatic children with and without caries were broadly similar. However, specific taxa, especially *Veillonella*, were significantly more abundant in caries-affected children, indicating a potential role in caries development. The study highlights that asthmatic children’s oral cavities may harbor opportunistic bacteria relevant to both caries and respiratory diseases, warranting further investigation into shared microbiome-pathogenesis pathways.
Wang 2023 [48]	To characterize changes in the oral (tongue dorsum) microbiome of older, long-term care patients receiving nutrition through nasogastric (NG) tubes, and to identify microbial taxa associated with potential aspiration pneumonia risk.	Design: Observational, cross-sectional study. Participants: 53 elderly patients in long-term care: 27 with NG-tube feeding 26 with oral feeding Sampling: Tongue swabs; exclusion of recent antibiotic users Microbial Analysis: 16S rRNA amplicon sequencing (V3–V4 regions); ASV-based bioinformatics Statistical Methods: Alpha/beta diversity metrics, hierarchical clustering, LEfSe to identify biomarkers, and co-occurrence network analysis	Diversity & Composition: Significant separation in microbiome composition between NG-tube and oral-feeding groups Longer duration of NG-tube placement correlated with distinct microbial profiles Biomarkers: NG-tube group: Enriched with Gram-negative aerobes like *Pseudomonas* and opportunistic *Corynebacterium* species (e.g., *C. striatum*, *C. casei*, *P. aeruginosa*, *P. stutzeri*). Oral-feeding group: Enriched with commensal anaerobes such as *Streptococcus* and *Veillonella* Co-occurrence Findings: Clear antagonistic relationship—pathogenic taxa (e.g., *Pseudomonas*, *Corynebacterium*) negatively correlated with commensals (*Streptococcus*, *Veillonella*). Pneumonia Association: Microbial signatures from NG-tube patients mirrored taxa associated with aspiration pneumonia events, supporting clinical relevance.	NG-tube feeding in older patients is associated with oral dysbiosis, shifting microbiome to a more pathogenic profile. Enrichment with opportunistic pathogens (*Pseudomonas*, *Corynebacterium*) and reduction of commensals may increase aspiration pneumonia risk. Microbiome data could inform targeted antimicrobial therapy prior to culture results. While causality cannot be claimed, findings are consistent across cultures and countries, suggesting predictable microbiome shifts linked to NG-tube placement.
Ucuncu 2024 [14]	To determine whether adults with asthma or COPD exhibit increased susceptibility to dental caries by analyzing saliva’s physical, chemical, and microbiological characteristics—focusing on the impact of respiratory disease medications on oral health.	Design: Cross-sectional comparative study. Participants (*n* = 104, aged 18–70): Asthma group (*n* = 41) Healthy controls (*n* = 21—inferred total) Oral Assessments: Dental caries via DMFT and DMFS indices Oral hygiene via Green & Vermillion Simplified OHI-S Saliva Analysis: Flow rate and buffering capacity Microbial counts: *Streptococcus mutans*, *Lactobacillus casei*, *Staphylococcus aureus*, and *Candida albicans* colony counts Caries Risk Profiling: Utilized the Cariogram software (https://cariogram.se; accessed on 1 January 2025) to integrate clinical and microbiologic risk factors	The respiratory disease groups (asthma + COPD) demonstrated significantly higher: DMFT, DMFS, and OHI-S scores compared to controls (*p* < 0.01) Cariogram-predicted caries risk also significantly elevated (*p* < 0.01), with no difference between asthma and COPD patients No significant difference in caries risk was found between COPD patients using two vs. three inhaled medications (*p* > 0.05)	Adults with asthma or COPD are more prone to dental caries, likely due to medication-induced changes in saliva and oral microbial environment. These individuals should adopt enhanced oral hygiene measures and undergo regular dental evaluations to mitigate heightened caries risk.
Al-Fahham 2025 [49]	To investigate the presence and virulence of Klebsiella pneumoniae in patients with oral cavity infections and to detect the fimH gene, which is associated with biofilm formation and pathogenicity.	Study Design: Cross-sectional, laboratory-based analysis. Samples: 150 oral swabs from patients (aged 7–65) with gingivitis, dental caries, and dental plaque, collected between September 2023–April 2024. Culture & Identification: Growth on blood and MacConkey agar. Biochemical tests (oxidase, indole, citrate, urease, TSI). Identification confirmed using VITEK 2 system. Antibiotic Sensitivity: Kirby-Bauer disk diffusion against 14 antibiotics. Biofilm Testing: Microtiter plate method (OD at 630 nm). Molecular Detection: PCR assay for fimH gene; electrophoresis for band size confirmation.	Bacterial Distribution: 82.7% of samples showed growth; 77.3% were Gram-negative. *K. pneumoniae* detected in 14/116 Gram-negative isolates (12.1%). Antibiotic Resistance (*K. pneumoniae*): High resistance to ticarcillin and piperacillin-tazobactam (88.8% each). Moderate resistance to trimethoprim-sulfamethoxazole (44.4%). No resistance to meropenem, imipenem, levofloxacin, ciprofloxacin, amikacin, or tobramycin. Biofilm Production: 85.7% of *K. pneumoniae* isolates were strong biofilm formers. 57.1% were encapsulated (capsule presence confirmed via Indian ink stain). Molecular Findings: fimH gene detected in 11/14 (78.8%) *K. pneumoniae* isolates. The gene is linked to type 1 fimbriae, which enhance adhesion and biofilm development.	There is a significant presence of antibiotic-resistant *K. pneumoniae* in the oral cavity of patients with dental infections in Al-Najaf. The fimH gene was prevalent in the majority of isolates, supporting its role in biofilm formation and virulence.
Cieplik 2020 [50]	To investigate associations between oral health, oral microbiota profiles, and the incidence of stroke-associated pneumonia (SAP) in patients admitted with acute stroke-like symptoms.	Design: Prospective observational cohort over 5 months (February–July 2018) Enrolled patients with stroke-like symptoms within 24 h of admission. Participants: 99 patients total—57 confirmed stroke, 42 stroke mimics. Timepoints: 1. Baseline (≤24 h): demographics, neurology, immunology (e.g., CRP), dental exam (DMFT), and microbiological sampling (saliva + subgingival plaque). 2. 48 h and 120 h: repeated immunology and microbiota sampling Microbial Analysis: Culture + 16S rRNA sequencing. Primary Outcome: Incidence of SAP in stroke patients, defined by clinical criteria. Exclusions: Intubated on admission, endocarditis prophylaxis, recent stroke (<1 month).	Incidence of SAP: 8 out of 57 stroke patients (14%) developed SAP. Risk Associations: SAP was significantly linked to older age, dysphagia, higher stroke severity (NIHSS), embolectomy, nasogastric tube placement, and elevated baseline CRP Oral Health: Trends towards more missing teeth and poorer oral hygiene in SAP patients, though differences were not statistically significant. DMFT scores similar across groups (~23–25). Microbiota Findings: No major differences in microbial composition between SAP and non–SAP groups. However, SAP patients exhibited ecological shifts over time—likely due to antibiotic usage.	SAP incidence (14%) in this stroke cohort aligns with previously reported ranges. Key SAP risk factors: age, dysphagia, stroke severity, nasogastric feeding, and inflammation (elevated CRP). Oral hygiene factors (e.g., tooth loss, plaque) showed a trend but did not reach statistical significance—likely due to small sample size. Oral microbiota composition wasn’t directly linked to SAP, but microbiome disruption post-SAP reflected antibiotic effects. Clinical recommendation: Larger studies needed to validate oral health strategies for SAP prevention and to assess implementing oral care protocols in stroke units.
Wang 2022 [51]	The aim of this study was to examine the impact of nasogastric tube feeding on the composition of the oral (tongue) microbiome in long-term care patients.	Tongue swab samples from 27 NG-tube-fed patients and 26 orally fed controls were analyzed using 16S rRNA next-generation sequencing to compare microbial composition.	NG-tube-fed patients had significantly altered oral microbiomes, with increased Gram-negative aerobes and higher levels of pneumonia-associated pathogens such as *Corynebacterium* and *Pseudomonas*, along with reduced levels of beneficial commensals like *Streptococcus* and *Veillonella*.	This study highlights distinct microbial changes in NG-tube-fed patients, offering important insights for improving oral care in long-term clinical settings.
Bairappan 2020 [52]	to investigate differences in salivary properties and oral health between adolescents with and without asthma, and to examine how asthma and its treatment influence the occurrence of dental caries	A cross-sectional study was conducted among 50 asthmatic and 50 non-asthmatic adolescents (12–15 years) in Bangalore. Salivary parameters and oral health were assessed using questionnaires, saliva analysis, and WHO 2013 criteria. Statistical tests were performed with significance set at *p* < 0.05.	Asthmatic adolescents had significantly more dental caries, gingival bleeding, and erosion than non-asthmatics. They also showed lower salivary flow, pH, buffering capacity, and higher levels of *S. mutans* and *Lactobacilli*. These factors were strongly linked to asthma severity and medication use.	Asthmatic adolescents demonstrated notably poorer salivary parameters and oral health. Both asthma and its treatment significantly influenced salivary function and increased the risk of dental caries in this group.
Willis 2021 [53]	The aim of this study was to characterize the composition of the oral microbiome in individuals with cystic fibrosis and to explore its potential role in respiratory health.	Oral rinse samples were obtained from 31 individuals with cystic fibrosis and matched controls in Spain. Bacterial and fungal communities were analyzed using 16S rRNA sequencing, culturing methods, and proteomics-based fungal identification.	The oral microbiome in CF patients showed reduced diversity, increased *Candida albicans* prevalence, and altered bacterial profiles linked to lung infections and oral diseases like caries and periodontitis.	This study offers an initial overview of the oral microbiome in cystic fibrosis, highlighting the need for further research into its connection with the lung microbiome.
Pinheiro 2021 [54]	The aim of this study was to investigate the composition of the oral and tracheal microbiota in children admitted to the pediatric intensive care unit.	An exploratory study was conducted among PICU patients aged 5 months to 13 years. Oral and tracheal samples were collected within the first 24–48 h of admission. Caries experience and oral hygiene were assessed using the DMFT/dmf and visual plaque index.	The mean DMFT/dmf score was 1.66, and the average visual plaque index was 43%. *Klebsiella pneumoniae* was the most frequently detected microorganism. Patients on mechanical ventilation had significantly higher rates of oral colonization by opportunistic pathogens compared to those breathing spontaneously. No significant association was found between plaque index or caries experience and changes in oral microbiota.	Children in intensive care are vulnerable to early colonization by respiratory and opportunistic pathogens, regardless of oral hygiene or dental condition.
Fourrier 1998 [55]	The influence of dental plaque decontamination on its colonization with aerobic bacteria of hospital infections in intensive care unit patients.	Sixty patients were divided into two groups. In the study group, a gel containing 0.2% CHX was applied to dental plaque three times daily. In both groups, bacterial cultures were performed from dental plaque, nasal swabs, tracheal aspirates, blood, and urine on days 0, 5, and 10, and weekly thereafter.	After 24 days, 50% of patients in the control group had at least one nosocomial pathogen in their dental plaque, compared with 28% in the study group. In tracheal aspirates, the figures were 45% and 28%, respectively.	The use of chlorhexidine gel may reduce the risk of hospital-acquired infections in intensive care unit patients.
Ortega 2015 [56]	Understanding the pathogenesis of aspiration pneumonia in different phenotypes of frail older patients with oropharyngeal dysphagia (OD).	Bacterial colonies of the oral/nasal cavity of 61 patients aged 70 years or older were assessed by PCR.	In the nasopharynx the predominant bacteria were: Moraxella, Corynebacterium, Staphylococcus, Streptococcus, and Alloiococcus, while in the oral cavity the predominant bacteria were: Veionella, Neisseria, Prevotella, Porphyromonas, Haemophilus, and streptococci.	Older, frail patients with OD have been found to have high oral bacterial concentrations and frequent colonization of the oral cavity with respiratory pathogens.
Fourrier 2000 [57]	Assessment of dental plaque colonization by aerobic bacteria and their association with nosocomial infections in intensive care unit patients.	Fifty-seven patients were examined every five days until discharge or death. The DMFT index and bacterial counts in dental plaque, nasal secretions, and tracheal aspirates were assessed. Cultures were performed on blood plates for 72 h.	The DMFT index remained stable throughout the stay. Aerobic bacterial colonies were present in 23% of patients on day 0 and in 46% of patients on day 10. Twenty-one patients developed nosocomial infections, of which six had the causative bacteria initially isolated from dental plaque.	Aerobic bacteria from dental plaque can be a significant cause of nosocomial infections in intensive care unit patients.
Varzhapetian 2019 [58]	Investigation of bacterial flora in iatrogenic maxillary sinusitis of dental origin.	From eight patients material was taken directly from the maxillary sinus wall during surgery. Cultures were incubated on 5% blood agar, boiled blood agar, Endo agar, and Chistovich’s medium up to 120 h.	Staphylococcus aureus (+) was identified in four patients. Additionally, staphylococcus epidermidis (+), Streptococcus pneumoniae (+), and Candida albikans were present in two patients each.	In iatrogenic maxillary sinusitis, which developed during the treatment of complications of maxillary caries, aerobic bacteria are cultured in 100.0% of patients and are represented by Gram-positive flora of Staphylococcus and Streptococcus (80.0%) and Candida fungi (20.0%).

## Supplementary Materials

The following supporting information can be downloaded at: https://www.mdpi.com/article/10.3390/jcm14217670/s1, Supplementary Table S1. Detailed characteristics of included studies; Supplementary Table S2. Quality assessment of included studies.
