# Supplementary material for: The Role of the Oral Microbiome and Dental Caries in Respiratory Health: A Systematic Review
Source: J Clin Med. 2025 Oct 29;14(21):7670. doi: 10.3390/jcm14217670 (PMC12608114; doi:10.3390/jcm14217670)
Supplement: Supplementary file 1 [file jcm-14-07670-s001.zip › Supplementary Table S1.pdf]

This table presents the detailed characteristics of included studies, applied in the systematic review titled: **The Role of the Oral Microbiome and Dental Caries in Respiratory Health: A Systematic Review**

Łukasz Zygmunt [1,*], Sylwia Kiryk [2], Kamil Wesołek [3], Jan Kiryk [4], Izabela Nawrot-Hadzik [5], Zbigniew Rybak [6], Klaudia Sztyler [2], Agata Małyszek [7], Jacek Matys [4,*] and Maciej Dobrzyński [2]

[1] Faculty of Medicine, Wroclaw University of Science and Technology, building C-7, pl. Grunwaldzki 11, 50-377 Wroclaw, Poland; lukasz.zygmunt@pwr.edu.pl (Ł.Z.)
[2] Department of Pediatric Dentistry and Preclinical Dentistry, Wroclaw Medical University, Krakowska 26, 50-425 Wroclaw, Poland; s.roguzinska@gmail.com (S.K.); maciej.dobrzynski@umw.edu.pl (M.D.); klaudia.sztyler@umw.edu.pl (K.S.)
[3] Józef Struś Multi-Specialty Municipal Hospital with a Care and Treatment Facility. Independent Public Health Care Facility, Szwajcarska 3, Poznan, Poland; wesolekwesolek@o2.pl (K.W.)
[4] Dental Surgery Department, Wroclaw Medical University, Krakowska 26, 50-425 Wroclaw, Poland; jan.kiryk@umw.edu.pl (J.K.); jacek.matys@umw.edu.pl (J.M.)
[5] Department of Pharmaceutical Biology and Biotechnology, Faculty of Pharmacy, Wroclaw Medical University, 50-556 Wroclaw, Poland izabela.nawrot-hadzik@umw.edu.pl (I.N-H.)
[6] Pre-Clinical Research Centre, Wroclaw Medical University, Bujwida 44, 50-345 Wroclaw, Poland; zbigniew.rybak@umw.edu.pl (Z.R.)
[7] Department of Biostructure and Animal Physiology, Wrocław University of Environmental and Life Sciences, Kożuchowska 1, 51-631 Wrocław, Poland; agata.malyszek@upwr.edu.pl (A.M.)
* Correspondence: jacek.matys@umw.edu.pl, lukasz.zygmunt@pwr.edu.pl

Supplementary Table S1. Detailed characteristics of included studies.

Study	Study Design and Population	Oral health status	Respiratory outcomes	Microbiological analysis—oral mouth	Microbiological analysis—respiratory tract	Key Findings / Associations
Zhou 2018 [16]	prospective, longitudinal, observational study 288 four-year-olds (155 boys, 133 girls) in Hong Kong	dmft and plaque index – mean dmft 2.1; PI 1.5	Number of upper respiratory tract infections (URI) episodes in 12 months 1 episode 47.9% 2 episodes 21.9% 3 or more 26%	qPCR from swabs: growth of Haemophilus and S. aureus in children with low dmft	No microbiological assessment	An inverse correlation was shown: dmft ↔ URTI count

Winning 2023 [42]	Cross-sectional study within the PRIME cohort. 507 men (aged 50–60 from the Belfast region)	Average 12 pockets PD≥4mm, 4 pockets ≥5mm; average CAL 3.2mm; subgingival smears	% predicted FEV_1 25,5% – 147,7%.	16S rRNA; in severe periodontitis ↑ Porphyromonas, ↓ α--diversity	No microbiological assessment	Advanced periodontal pockets correlate with significant deterioration of FEV_1, suggesting the impact of periodontal dysbiosis on respiratory function.
Rantala 2016 [43]	Prospective population-based cohort study 1623 children born between 1984–1990	Number of filled permanent teeth (FT) Mean 3.5	LRTI requiring hospitalization by age 7.	No microbiological assessment	No microbiological assessment	LRTI up to 2 years of age → +1.5 FT; LRTI 2–7 years of age → +1.2 FT. Early LRTI predicted more restorations in adults.
Shiraziian 2018 [44]	case -control study 42 patients with acute or chronic lung diseases (15–65 years old)	Gingivitis (0–3): 69% of patients had a score of 3 vs. 7% of controls Periodontitis (4–6 mm CAL): 36% of patients had a score of 5–6 vs. 12% of controls Halitosis (2–4): 71% of patients had a score of ≥2 vs. 28% of control Caries: 93% of patients vs. 88% of controls Xerostomia & oral lesions	History of lung disease Lack of precise data	No microbiological assessment	No microbiological assessment	Significantly worse periodontal status and halitosis in patients with lung diseases vs. controls, regardless of disease severity; suggests the need to improve oral hygiene in pulmonary patients.
Ploenes 2022 [45]	Prospective, observational study 230 adult patients (median age 64 years, 56% men) qualified	Regular dental visits: 38.3% Tooth loss: 90% of patients Active caries: 61.7%	FEV_1% predicted mean = 84,2% ± 22,1, occurrence of postoperative complications	No microbiological assessment	Tracheal aspirate cultures in two infections: P. aeruginosa	Active oral caries is a significant risk factor for postoperative complications, especially postoperative pneumonia.

	for elective thoracic surgery in Essen	Periodontal disease: 82.6% (40% chronic, 42.6% progressive/advanced)	= 30.4% of patients			
Arweiler 2021 [46]	cross-sectional study Children aged 6-16 years with allergic asthma or atopic without asthma	SFR (mL/min): AA 0,33±0,18; AT/AL 0,53±0,25; control 0,28±0,16<br><br>dmft/DMFT: 0,47±1,30; 1,00±2,00; 1,27±1,91<br>PSI: 0,73; 0,41; 1,07<br>GBI (%): 16±13; 8±9; 36±12<br>PCR (%): 68±27; 65±27; 51±11	Presence of AA--Chd or AT/AL--Chd	16S rRNA NGS (V4); biofilm β-diversity: AA & AT/AL similar, both different from Control; taxa in biofilm in AA/AT: ↑Capnocytophaga spp., Fusobacterium, Prevotella_6, Mannheimia; ↓Cardiobacterium hominis, Fusobacterium nucleatum, Haemophilus sp.	No microbiological assessment	Allergic/asthmatic children have a clearly altered biofilm composition vs healthy children, suggesting a role of dental biofilm in the pathogenesis of allergy.
Bellissimo-Rodrigues 2014 [41]	Participants: 254 adult ICU patients expected to stay ≥48 h<br>Groups: Experimental group (n = 127): Received dental care by a dental surgeon<br>Control group (n = 127): Received standard nurse-delivered oral hygiene<br>Exclusion criteria: Pregnancy, blood dyscrasias<br>Baseline conditions: Similar between groups, with	Method: Clinical oral health exam<br>Parameters Assessed: Oral Hygiene Index (OHI-S) (WHO criteria)<br>Edentulism, caries, residual roots, gingival inflammation, periodontal pockets, mucositis, candidiasis<br>Findings: Similar oral health status between groups<br>OHI-S better in the intervention	Primary Outcome: Incidence of lower respiratory tract infections (LRTIs)<br>LRTI incidence: 8.7% in dental care vs. 18.1% in control (Adjusted RR = 0.44; *p* = 0.04)<br>VAP incidence: 7.6 vs. 16.5 per 1,000 ventilator-days (*p* < .05)<br>Mortality: No significant difference; trend toward	(no sequencing or cultures performed on oral cavity)	No statistically significant differences in pathogen distribution between groups<br>Cultures positive in 86.5% of LRTI cases<br>Of the 34 patients who developed LRTIs: 86.5% (n = 30) had a pathogen identified through cultures.<br>1. Non-fermentative Gram-negative bacilli<br>2. Enterobacteriaceae	Professional dental care significantly reduced LRTI incidence (RR = 0.44, 95% CI: 0.20–0.96).<br>Greatest impact seen in reduced VAP incidence (cut by over 50%).<br>Oral hygiene improvement (fewer periodontal pockets and better OHI-S) was statistically linked to reduced infection rates.<br>Although not directly proven by microbial sequencing, the

	slight differences in age and obesity prevalence	group (mean 1.96 vs. 2.33) Edentulism and periodontal pockets were more frequent in control group	fewer LRTI-related deaths in intervention group		3. Gram-positive cocci 4. Candida	removal of oral infection foci is strongly implicated in reducing pathogen colonization of the lower airways. Microbial patterns in the lungs reflect typical hospital-acquired and opportunistic pathogens, some of which can originate from oral reservoirs.
Cherkasov 2019 [47]	Type: Cross-sectional observational comparative study<br><br>Duration: 8 weeks in August-September 2016<br><br>Setting: Centre of Paediatric Allergology and Clinical Immunology, Regional Hospital No. 2, Orenburg, Russia<br><br>Focus: Comparing dental plaque microbiota in asthmatic children with and without dental caries	Methods: WHO criteria for dental caries diagnosis No Examiner: Single trained examiner<br><br>Caries definition: Lesion with detectably softened floor, undermined enamel, or softened wall<br><br>Index: dmft (decayed, missing, filled teeth)<br><br>Classification: Binary (1 = caries present, 0 = caries absent)<br>Key findings:	Primary condition:<br><br>Bronchial asthma (all participants)<br><br>Assessment: GINA criteria for severity classification<br><br>Status: Acute exacerbation at admission<br><br>Respiratory pathogen identification: Through oral microbiome analysis<br><br>Limitations: No direct respiratory tract sampling No specific respiratory tract	Sampling sites: Group A: Second deciduous mandibular left molar (75) Group C: Most affected tooth with largest cavitated lesion Collection method: Sterile metal spoon excavator Timing: Morning, 12h after evening tooth brushing, fasting condition Storage: DNA-Express buffer, frozen at -20 °C Sequencing: Target: 16S rRNA gene V3-V4 regions Platform: Illumina MiSeq, 2×300bp paired-end	Not performed	Sequencing Results High-quality data: Good's coverage >99.5% for both groups<br><br>Comprehensive diversity: 77 species, 37 genera, 25 families identified<br><br>No significant alpha diversity differences between groups Taxonomic Composition Major phyla: Firmicutes, Proteobacteria, Actinobacteria, Bacteroidetes, Fusobacteria<br><br>Common genera (both groups): Streptococcus, Neisseria,

	Total sample size: 18 children aged 3-6 years<br>All participants: Admitted with acute asthma exacerbation<br>Group allocation:<br>Group A (asthma without caries): 8 children (6 boys, 2 girls)<br>Group C (asthma with caries): 10 children (4 boys, 6 girls)<br>Demographics:<br>Age range: 3-6 years<br>Group A: mean 55±11 months<br>Group C: mean 53±12 months<br>No significant age/gender differences	Group A: dmft = 0 for all children<br>Group C: dmft range 1-8 (mean 2.9±0.7)<br>Clear separation between groups	infections assessed<br>Respiratory connection inferred from literature	Processing: USEARCH pipeline with 97% similarity clustering<br>Quality control: Comprehensive coverage (>99.5%)		Veillonella, Prevotella, Haemophilus<br><br>Group-specific genera:<br>Caries-affected only: Fusobacterium<br>Caries-free only: Corynebacterium, Kingella<br>Differential Abundance Analysis<br>"Caries-enriched" taxa ($p<0.05$):<br><br>Genus: Veillonella (most important finding)<br><br>Higher taxonomic levels: Negativicutes (class), Selenomonadales (order), Veillonellaceae (family)<br><br>Species: Prevotella loescheii, P. histicola, Kingella oralis, Haemophilus haemolyticus<br><br>"Caries-depleted" taxa ($p<0.05$):<br>Genus: Neisseria<br>Species: Neisseria oralis

Caries-Microbiome Relationships:<br>-Veillonella significantly higher in caries-affected children<br>-Consistent with literature on Veillonella in cariogenic biofilms<br>-Expanded view of cariogenic community beyond traditional S. mutans/S. sobrinus<br>Respiratory Pathogen Implications:<br>-Multiple oral taxa identified as potential respiratory pathogens<br>-Present in both caries-affected and caries-free asthmatic children<br>-Oral-respiratory pathogen overlap:<br>Veillonella: Cariogenic + found in ventilated patients<br>Haemophilus: Respiratory pathogen + dental plaque colonizer<br>Fusobacterium, Prevotella, Porphyromonas: Periodontal + pneumonia pathogens

Novel Findings<br>Low abundance of classical cariogenic bacteria (S. mutans, S. sobrinus)<br><br>Veillonella as key caries-associated genus in asthmatic children<br><br>Evidence for oral cavity as respiratory pathogen reservoir
High Relevance Factors<br><br>Direct comparison of caries vs non-caries microbiomes in respiratory disease<br><br>Clear caries assessment with standardized WHO criteria<br><br>Respiratory disease context—all participants asthmatic<br><br>Strong microbiome methodology—comprehensive 16S rRNA sequencing<br><br>Statistical significance in key findings

						Specific Contributions<br><br>Identifies Veillonella as caries-enriched genus with respiratory pathogen potential<br><br>Demonstrates oral-respiratory pathogen overlap in clinical population<br><br>Provides evidence for oral cavity as respiratory pathogen reservoir<br><br>Shows caries-specific microbiome changes in respiratory disease context
Wang 2023 [48]	Type: Cross-sectional observational comparative study<br>Duration: March to October 2021<br>Setting: Yilan County, Taiwan, and National Yang Ming Chao Tung University Hospital<br>Comparison groups: Nasogastric tube feeding vs.	Methods: Full-mouth oral examination by dentist<br>Parameters measured: Dental caries assessment<br>DMFT-related indices<br>Gingival status (healthy, mild gingivitis, severe gingivitis)<br>Residual root assessment	Primary outcome: History of pneumonia (questionnaire-based)<br>NG-tube group: 63% pneumonia history<br>Oral-feeding group: 12% pneumonia history<br>Secondary focus: Aspiration pneumonia risk assessment	Sampling site: Tongue dorsum<br>Collection method: Sterilized cotton swabs stored in TE buffer<br>Processing: DNA extraction: AllPure Bacteria Genomic DNA Kit<br>Sequencing: Illumina MiSeq platform, 150-bp paired-end	NOT PERFORMED	Microbial Composition Differences<br>Significant differences in tongue microbiome between groups (p<0.00001)<br>Nearly complete separation in hierarchical clustering<br>No significant differences in alpha diversity<br>NG-tube Associated Pathogens (Enriched)

	oral feeding patients<br>sample size: 53 participants<br>NG-tube group: 27 patients<br>Oral-feeding group: 26 patients<br>Demographics:<br>Mean age: 73.92±22.74 years (NG-tube) vs. 65.76±16.77 years (oral-feeding)<br>Gender distribution: NG-tube (8M/19F), Oral-feeding (14M/12F)<br>Bedridden status: 96% (NG-tube) vs. 31% (oral-feeding)	Dental prosthetics evaluation<br>Key findings: NG-tube patients showed worse oral health with higher caries index, more residual roots, and more severe gingivitis (85% vs. 54%)	Limitations: No direct measurement of current respiratory infections or lower respiratory tract sampling	Target: 16S rRNA V3-V4 regions<br>Primers: 341F and 805R<br>Analysis:<br>Pipeline: QIIME2 with DADA2 algorithm<br>Taxonomic classification: SILVA database<br>Total ASVs detected: 5,480<br>Read counts: ~78,867±38,367 (NG-tube), ~77,290±39,006 (oral-feeding)		Pseudomonas (P. aeruginosa, P. stutzeri, P. otitidis)<br>Corynebacterium (C. striatum, C. casei, C. simulans)<br>Parvimonas, Fusobacterium, Actinotignum, Mycoplasma<br>Characteristics: More Gram-negative aerobes, opportunistic pathogens<br>Oral-feeding Associated Commensals (Enriched)<br>Streptococcus (S. sanguinis, S. mitis, S. salivarius)<br>Veillonella (V. atypica, V. rogosae)<br>Leptotrichia, TM7×, Actinomyces, Rothia, Prevotella<br>Characteristics: Mostly Gram-positive anaerobes, normal oral flora<br>Clinical Associations<br>Strong correlation between NG-tube duration and microbiome changes (p=0.00003)<br>Pneumonia history significantly associated with

						feeding method (p=0.00028) Negative correlation between pathogenic and commensal species Symbiotic relationship between Streptococcus and Veillonella Clinical Implications NG-tube associated pathogens are known aspiration pneumonia causative agents -Potential for microbiome-based risk stratification -May inform empiric antimicrobial therapy selection
Ucuncu 2024 [14]	Type: Cross-sectional comparative observational study Objective: To assess the association between chronic respiratory diseases (asthma and COPD) and dental caries risk, focusing on changes in saliva and oral microbial load. Total participants:	Clinical examination parameters: Salivary flow rate Cariogram: Median for: asthma 49, COPD 51, control 71 DMFT index (Decayed, Missing, Filled Teeth) DMFS index (Decayed, Missing, Filled Surfaces) OHI-S (Simplified	No acute respiratory events were assessed. Chronic conditions were: Asthma patients using inhaled corticosteroids (ICS) and β2-agonists COPD patients using long-acting β2-agonists, anticholinergics, and sometimes corticosteroids	Sample type: Unstimulated saliva samples Tests conducted: Microbial culture and colony counting of *Streptococcus mutans, Lactobacillus casei, Staphylococcus aureus, Candida albicans* Results: Higher colony counts of *S. mutans*, *L. casei*, and *C. albicans* in COPD and	No lower respiratory tract samples	All respiratory disease groups had significantly higher microbial loads than controls for all taxa analyzed. Patients with COPD and asthma had: Significantly higher caries indices (DMFT/DMFS) Worse oral hygiene (OHI-S) Higher salivary counts of cariogenic and opportunistic organisms

	104 adults (aged 18–70 years) COPD group: 42 patients Asthma group: 41 patients Control group: 21 healthy individuals	Oral Hygiene Index) Statistically significant differences were found between the respiratory disease groups and controls in all indices ($p < 0.01$) DMFT median 14(asthma), COPD (20), control (7)	No pneumonia or VAP was evaluated, as the study focused on stable outpatients	asthma groups than in controls ($p < 0.01$) Reflects an acidogenic and cariogenic shift in oral microbiota in respiratory disease patients Reflects an acidogenic and cariogenic shift in oral microbiota in respiratory disease patients Statistical analysis (NCSS—Number Cruncher Statistical System)		Inhaled medications, especially corticosteroids, likely contribute to salivary flow reduction and microbiome dysbiosis
Al-Fahham 2025 [49]	Study Design Type: Cross-sectional observational laboratory-based study Duration: September 2023 to April 2024 Setting: Iraq Focus: Molecular detection of fimH gene from Klebsiella pneumoniae isolated from oral cavity patients Population Total sample size: 150 patients with oral cavity diseases Target analysis: 14 K.	Oral Health Assessment Methods: Clinical diagnosis of oral conditions Parameters assessed: Gingivitis (clinical diagnosis) Dental caries (clinical diagnosis) Dental plaque (clinical diagnosis) Sample collection: Swabs from oral infection sites after tooth removal Limitations: No standardized	NO RESPIRATORY OUTCOMES ASSESSED	Microbiome Analysis—Oral/Mouth Sampling method: Swabs from oral infection sites Transport: Special transport media Culture methods: Blood agar and MacConkey agar Positive cultures: 124/150 (82.7%) Identification methods: VITEK-2 compact system for Gram-negative bacteria Conventional biochemical	NOT PERFORMED	Bacterial Distribution in Oral Cavity Total positive cultures: 124/150 (82.7%) Gram-negative bacteria predominant: 116/124 (93.5%) Enterobacteriaceae: 72/116 (62%) of Gram-negative bacteria Major oral bacterial isolates: K. pneumoniae: 14 isolates Enterobacter cloacae: 12 isolates Pseudomonas aeruginosa: 10 isolates E. coli: 9 isolates Distribution by Oral Condition

	pneumoniae isolates<br>Demographics:<br>Age range: 7 to 65 years<br>Gender distribution: 70 males, 80 females<br>Clinical conditions:<br>Gingivitis<br>Dental caries<br>Dental plaque<br>Inclusion criteria:<br>Patients with oral cavity infections aged 7-65 years<br>Diagnosed with gingivitis, dental caries, and/or dental plaque	oral health indices (DMFT, DMFS)<br>No detailed oral examination methodology<br>Focus primarily on microbiological identification		tests (oxidase, motility, IMVIC tests)<br>Gram staining<br>Target focus: Klebsiella pneumoniae specifically<br>Results:<br>K. pneumoniae isolation: 14/116 (12.1%) of Gram-negative bact.<br>Total K. pneumoniae: 14/150 (9.3%) of all specimens<br>Molecular Analysis<br>Target gene: fimH gene (encodes Type 1 fimbriae)<br>Method: Conventional PCR<br>Results: 11/14 (78.8%) K. pneumoniae isolates positive for fimH gene		K. pneumoniae found equally across conditions:<br>Gingivitis: 5 isolates<br>Dental caries: 4 isolates<br>Dental plaque: 5 isolates<br>Antibiotic Resistance Pattern<br>High resistance rates:<br>Ticarcillin: 88.8%<br>Piperacillin-tazobactam: 88.8%<br>Moderate resistance:<br>Trimethoprim-sulfamethoxazole: 44.4%<br>Cefepime: 33.3%<br>No resistance observed:<br>Carbapenems (Meropenem, Imipenem): 0%<br>Fluoroquinolones: 0%<br>Amikacin, Tobramycin: 0%<br>Virulence Factors<br>fimH gene: 78.8% positive (11/14 isolates)<br>Biofilm formation: 85.7% strong biofilm formers (12/14)<br>Capsule production: 57.1% encapsulated (8/14)<br>Association: fimH gene linked to biofilm

						formation capabilities<br>K. pneumoniae present across all oral infection types<br>High prevalence of multidrug resistance<br>Significant virulence factor expression<br>Potential for biofilm-mediated treatment resistance
Cieplik 2020 [50]	Type: Prospective observational cohort<br>Setting: Stroke unit<br>Duration: ~5 months (Feb–July 2018)<br>Timepoints: Baseline (≤24 h of admission), 48 h (follow--up), 120 h (final follow--up) for repeated sampling/microbiota and immunologic evaluation<br>Population: 99 patients with stroke-like symptoms (57 strokes, 42 mimics)<br>Median age 66 (stroke mimics) (-79,5 stroke with pneumonia)	Clinical parameters recorded at baseline:<br>DMFT index: median scores between ~23–25 across groups (no significant difference)<br>full-mouth approximal plaque index (API)<br>Probing pocket depths (PPDs) were measured at six points for each tooth—the deepest pocket (PPD_{max}) was recorded for each tooth	Primary outcome: Incidence of stroke-associated pneumonia (SAP) in confirmed stroke patients<br>Results: 8 of 57 (14%) developed SAP within the observation period<br>Significant risk factors: increased age (median 79,5)<br>Dysphagia (75%)<br>higher stroke severity (e.g., NIHSS 6.5 vs. 2), embolectomy (62,5%), nasogastric tube use (62,5%), elevated baseline CRP	Sample types: Tongue dorsa<br>Subgingival plaque (from baseline, 48 h, 120 h)<br>Methods: Culture-based analysis—MALDI-TOF MS<br>16S rRNA gene amplicon sequencing<br>Findings: No significant baseline differences in microbial composition between groups with and without SAP: *Rothia mucilaginosa* (detected in 76.8% of patients), viridans streptococci (75.8%), *Prevotella melaninogenica* (74.7%), *Streptococcus*	not conducted in this study	SAP occurred in 14% of stroke patients and was strongly associated with known clinical predictors (dysphagia, CRP, stroke severity)<br>Oral health factors (missing teeth, hygiene) had non-significant trends toward more risk in SAP cases<br>Oral microbiome composition at admission did not significantly differ between those who subsequently developed pneumonia and those who did not<br>A temporal shift in microbiota during hospitalization was observed in pneumonia patients, plausibly due to

				oralis (61.6%), and *Neisseria* spp. (59.6%) the stroke-associated pneumonia group was subject to some changes over time with ↓↓*Streptococcus, Neisseria, Prevotella, Rothia, Haemophilus*, and *Leptotrichia* spp. by >20% each. ↑↑*Staphylococcus*, *Klebsiella*, and *Candida* spp. increased by >20% each.		antibiotic therapy
Wang 2022 [51]	Observational, cross-sectional study<br><br>53 elderly individuals residing in long-term care facilities in Taiwan:<br>27 participants received nutrition via a nasogastric tube (NGT group),<br><br>26 participants were orally fed (control group).	Dry mouth (xerostomia),<br><br>Accumulation of oral biofilm, (tongue microbiome sampling)<br>Increased visible debris on the tongue surface.	The oral presence of known respiratory pathogens (Pseudomonas, Acinetobacter) suggests elevated risk for aspiration pneumonia, especially in patients with NGTs.	Method:<br>16S rRNA gene sequencing targeting the V3–V4 regions, using Illumina MiSeq technology.<br><br>Microbial DNA was extracted from tongue coating samples.<br><br>Main Findings:<br>Significantly reduced alpha diversity (lower species richness and Shannon index) in the NGT group.<br><br>NGT patients had higher abundance of Proteobacteria	No information	NG-tube feeding is associated with significant shifts in oral microbiome, characterized by:<br>Enrichment of opportunistic gram-negative aerobes (Pseudomonas, Corynebacterium), which are linked to pneumonia risk<br>Lower abundance of typical commensals such as Streptococcus and Veillonella<br>The findings imply a mechanistic link: Oral dysbiosis → Pathogen overgrowth →

				and genera such as: *Corynebacterium*, *Acinetobacter*, *Pseudomonas*, *Rothia*.<br><br>Depletion of commensal genera like: *Streptococcus*, *Veillonella*, *Prevotella*.<br><br>Dysbiotic oral environment, potentially favoring colonization by opportunistic and respiratory pathogens.		Higher risk of respiratory infection, particularly in the form of aspiration pneumonia or VAP (ventilator-associated pneumonia).
Bairappan 2020 [52]	cross-sectional comparative study<br><br>50 asthmatic and 50 nonasthmatic adolescents aged 12-15 years in Bangalore City India	DMFT index,<br><br>Gingival bleeding,<br><br>Dental erosion,<br><br>Fluorosis, traumatic injuries, mucosal lesions, and treatment needs	INDIRECT<br><br>inferred through elevated cariogenic bacterial counts (S. mutans, Lactobacilli) and salivary alterations linked to asthma medication use, which may elevate risk for oral–respiratory interplay	Parameters Assessed: - Salivary flow rate (unstimulated) - pH Buffering capacity - Salivary counts of Streptococcus mutans and Lactobacilli Results: Asthmatic adolescents showed significantly lower flow rate, pH, and buffering capacity, and elevated levels of S. mutans and Lactobacilli ($p < .05$)	NO	Asthmatic adolescents had worse oral health status, with higher caries prevalence, gingival bleeding, and dental erosion compared to controls ($p < .05$)<br><br>Salivary dysfunction associated with asthma and its treatment-reduced flow, lower pH, decreased buffering, along with elevated S. mutans and Lactobacilli counts, correlating significantly with increased

						caries experience (p < .001) Odds of poor salivary parameters and dental disease were multiple times higher in asthmatics: up to 6× higher for cariogenic bacteria, 3.6× for low pH
Willis 2021 [53]	Observational, cross-sectional 31 individuals with cystic fibrosis (CF), aged 7–47 years<br><br>Samples collected across Spain during January–November 2017, in collaboration with CF associations	INDIRECT<br><br>microbial diversity and potential oral--health implications, e.g., presence of *Candida albicans* and bacteria linked to periodontitis and dental caries	INDIRECT<br><br>dentification of potential airway pathogens such as *Pseudomonas* and increased presence of *Candida albicans* in the oral cavity of CF patients allows inference of elevated risk for respiratory colonization or infection, notably in the lower airways	16S rRNA metabarcoding (bacterial profiling) and high--throughput sequencing; fungal identification via culture and proteomics (Candida spp.) CF individuals demonstrated significantly lower alpha diversity (reduced species richness and Shannon/Simpson indices) compared to controls Elevated prevalence of genera with relevance to respiratory infections, including *Pseudomonas*, and shifts in oral taxa associated with periodontal/caries risk (e.g., periodontitis-	NO INFORMATION	CF was linked to marked oral dysbiosis, with lower bacterial diversity, significant shifts in community composition, and greater prevalence of fungal and bacterial taxa that may serve as reservoirs for pulmonary pathogens Candida albicans was notably more common in CF samples The microbiome changes observed suggest possible associations between altered oral ecology in CF and risk of respiratory disease.<br><br>Authors conclude that oral sampling may reflect lung microbial ecology in CF

				associated species more frequent in CF) *Candida albicans* was significantly more prevalent in CF subjects than matched controls		
Pinheiro 2021 [54]	Exploratory cross-sectional study<br><br>Pediatric and adolescent patients (age: 5 months to 13 years) admitted to a Pediatric Intensive Care Unit (PICU) in a hospital in Salvador, Brazil. Participants included both mechanically ventilated and spontaneously breathing patients.<br><br>Microbiological sampling was performed within 24 and 48 h of PICU admission	DMFT/dmft: 1.66 (SD = 2.18)<br><br>VPI(Visual Plaque Index): 43.03% (SD = 36.93%)	INDIRECT<br><br>early presence of potential pathogens such as K. pneumoniae in the oral cavity and tracheal secretions suggests elevated risk of nosocomial respiratory infection, especially in ventilated children	microbial culture techniques posterior tongue swabs (at 24 and 48h)<br><br>Klebsiella pneumoniae was the most frequently detected pathogen (15% prevalence) among oral isolates<br><br>Oral colonization with opportunistic pathogens was significantly more common in mechanically ventilated patients (p < .05)<br>DMFT/dmft and VPI scores didn't correlate with microbial changes (no significant association)	Additional sampling from tracheal secretions in mechanically ventilated patients within 48 h<br>Patterns mirrored those in oral samples: higher colonization by opportunistic respiratory pathogens.	Children in PICU—especially those under mechanical ventilation—are rapidly colonized by opportunistic respiratory pathogens like *Klebsiella pneumoniae*<br>There was no significant link between dental indices (DMFT/dmft, VPI) and microbial colonization.<br><br>vulnerability of pediatric ICU patients to early opportunistic colonization
Fourrier 1998 [55]	Single-blind randomized comparative study, ICU patients with	The Caries-Absent-Occluded index (CAO)—lack of precise data	VAP, bronchitis, bacteriemia Lack of precise data	Bacterial culture from dental plaque Presence of Pseudomonas, Acinetobacter,	Bacterial culture from tracheal aspirates and bronchial secretions	Using 0.2% CHX gel reduces the level of bacteria in dental plaque, which reduces the risk of

	mechanical ventilation	semi-quantitative plaque index mean before treatment = 2.2, after = 1.1		Enterobacter spp 29% of patients in the study group and 66% in the control group.	18,4% study group 17.5% control group	developing nosocomial infections.
Ortega 2015 [56]	cross-sectional observational studies 61 patients ≥70 years of age with oropharyngeal dysphagia	OHI--S 2.1–2.5; 90% had periodontitis; 72% caries; tongue-coating 30–45%	Aspiration pneumonia was diagnosed in 17 of the OD-APN group; delayed LVC (409±115ms) and 15.6% silent aspirations	qPCR: colonization with respiratory pathogens in 93% of OD, 67% of controls; pyrosequencing: oral rinse and saliva with ↑ Klebsiella, Pseudomonas and ↓ Streptococcus commensali	qPCR Total bacterial load: <10^6 CFU	FOP with OD have high oral colonization with respiratory pathogens (>90%), which together with delayed LVC and silent aspirations increases the risk of aspiration pneumonia.
Fourrier 2000 [57]	Prospective observational study, ICU patients	CAO index medium to high, Mean Semiquantitative dental plaque score = 2.5	Bacterial cultures from tracheal aspirates (TA), Monitoring of nosocomial infections	Bacterial cultures from dental plaque, increase in plaque during stay, presence of aerobic bacteria.	Quantitative cultures of TA and nasal secretions, increased colonization with respiratory bacteria, presence of identical strains in dental plaque and TA	Dental plaque is a reservoir of bacteria from where they can migrate to the respiratory tract and cause aspiration pneumonia.
Varzhapetian 2019 [58]	Prospective, clinical observational study, patients with maxillary sinusitis developed after treatment of caries complications, mean age 40.5	Material was collected directly from the maxillary sinus cavity during surgery (no oral samples were taken); the presence and number of bacterial and fungal colonies (CFU) were assessed.	Examination of aspirates taken from the maxillary sinus during surgery	Cultures directly from the sinus: 100% of patients $>10^3$CFU; Gram-positive Staphylococcus predominated (S. aureus 40%, S. epidermidis 20%) and Streptococcus pneumoniae 20%, Candida albicans 20%	Aerobic culture (Vitek 2 Compact, BioMérieux) + fungal culture and antibiotic resistance tests (Vitek) Staphylococcus and Streptococcus – 80%, Candida – 20%.	The presence of periapical lesions leads to the formation of oroantral connections, which allows the migration of microorganisms from the oral cavity to the maxillary sinus.
