# Supplementary material for: The Role of the Oral Microbiome and Dental Caries in Respiratory Health: A Systematic Review
Source: J Clin Med. 2025 Oct 29;14(21):7670. doi: 10.3390/jcm14217670 (PMC12608114; doi:10.3390/jcm14217670)
Supplement: Supplementary file 1 [file jcm-14-07670-s001.zip › Supplementary Table S2.pdf]

This table presents the quality assessment of included studies, applied in the systematic review titled: **The Role of the Oral Microbiome and Dental Caries in Respiratory Health: A Systematic Review**

Łukasz Zygmunt [1,*], Sylwia Kiryk [2], Kamil Wesołek [3], Jan Kiryk [4], Izabela Nawrot-Hadzik [5], Zbigniew Rybak [6], Klaudia Sztyler [2], Agata Małyszek [7], Jacek Matys [4,*] and Maciej Dobrzyński [2]

1 Faculty of Medicine, Wroclaw University of Science and Technology, building C-7, pl. Grunwaldzki 11, 50-377 Wroclaw, Poland; lukasz.zygmunt@pwr.edu.pl (Ł.Z.)
2 Department of Pediatric Dentistry and Preclinical Dentistry, Wroclaw Medical University, Krakowska 26, 50-425 Wroclaw, Poland; s.roguzinska@gmail.com (S.K.); maciej.dobrzynski@umw.edu.pl (M.D.); klaudia.sztyler@umw.edu.pl (K.S.)
3 Józef Struś Multi-Specialty Municipal Hospital with a Care and Treatment Facility. Independent Public Health Care Facility, Szwajcarska 3, Poznan, Poland; wesolekwesolek@o2.pl (K.W.)
4 Dental Surgery Department, Wroclaw Medical University, Krakowska 26, 50-425 Wroclaw, Poland; jan.kiryk@umw.edu.pl (J.K.); jacek.matys@umw.edu.pl (J.M.)
5 Department of Pharmaceutical Biology and Biotechnology, Faculty of Pharmacy, Wroclaw Medical University, 50-556 Wroclaw, Poland izabela.nawrot-hadzik@umw.edu.pl (I.N-H.)
6 Pre-Clinical Research Centre, Wroclaw Medical University, Bujwida 44, 50-345 Wroclaw, Poland; zbigniew.rybak@umw.edu.pl (Z.R.)
7 Department of Biostructure and Animal Physiology, Wrocław University of Environmental and Life Sciences, Kożuchowska 1, 51-631 Wrocław, Poland; agata.malyszek@upwr.edu.pl (A.M.)
* Correspondence: jacek.matys@umw.edu.pl , lukasz.zygmunt@pwr.edu.pl

Supplementary Table S2. Quality assessment of included studies.

Author	Is there an adequate rationale for using a mixed methods design to address the research question?	Are the different components of the study effectively integrated to answer the research question?	Are the outputs of the integration of qualitative and quantitative components adequately interpreted?	Are divergences and inconsistencies between quantitative and qualitative results adequately addressed?	Do the different components of the study adhere to the quality criteria of each methodological tradition involved?
Zhou 2018 [16]	yes	yes	yes	yes	yes
Winning 2023 [42]	Yes	yes	yes	yes	yes
Rantala 2016 [43]	Yes	yes	yes	yes	yes
Shirazian 2018 [44]	yes	yes	yes	yes	yes
Ploenes 2022 [45]	yes	yes	yes	yes	yes
Arweiler 2021 [46]	yes	yes	yes	yes	yes
Bellissimo-Rodrigues 2014 [41]	yes	yes	yes	yes	yes
Cherkasov 2019 [47]	yes	yes	yes	yes	yes
Wang 2023 [48]	yes	yes	yes	yes	yes
Ucuncu 2024 [14]	yes	yes	yes	yes	yes

Al-Fahham 2025 [49]	yes	yes	yes	yes	yes
Cieplik 2020 [50]	yes	yes	yes	yes	yes
Wang 2022 [51]	yes	yes	yes	yes	yes
Bairappan 2020 [52]	yes	yes	yes	yes	yes
Willis 2021 [53]	yes	yes	yes	yes	yes
Pinheiro 2021 [54]	yes	yes	yes	yes	yes
Fourrier 1998 [55]	yes	yes	yes	yes	yes
Ortega 2015 [56]	yes	yes	yes	yes	yes
Fourrier 2000 [57]	yes	yes	yes	yes	yes
Varzhapetian 2019 [58]	yes	yes	yes	yes	yes
